# Chronic Syphilitic Salivation

**Published:** 1890-06

**Authors:** 


### Chronic Syphilitic Salivation.

A. W. Furber, M.D., L.R.C.S., L.D.S., says: "I have for a long time had a gentleman patient under my care for disease of the teeth, and although my operations progressed favorably, I had many difficulties to contend with. The whole of my patient's teeth appeared to have syphilitic taint, and with increased flow of saliva amounting to chronic salivation. These were not the only troubles I had to surmount; but that which retarded my work most was the repeated recurrence of syphilitic ulcers of the sulcus and gums generally, which, though not painful to my patient, was still a source of considerable discomfort and militated greatly against the success of my operations. Iodia having come under my notice, I was inclined to give it a trial, and with the addition of a small proportion of liq. hydrarg. bichlor., taken daily before meals, for a time—also used occasionally as a mouth wash—the salivation became normal, the mucous membrane assumed a more healthy state, and the teeth generally looked like coming back to their original color."

80 Fortress Road, London, N W.

